# Supplementary material for: WordCommentsAnalyzer: A windows software tool for qualitative research
Source: F1000Res. 2018 Sep 4;7:536. Originally published 2018 May 3. [Version 2] doi: 10.12688/f1000research.14819.2 (PMC6137411; doi:10.12688/f1000research.14819.2)
Supplement: Supplementary file 3 [file f1000research-7-17614-s0002.tgz › b2a79496-4ada-47c4-9682-a99ce867836d.zip!/Eight Iranian Health Journals/Journal of Research in Rehabilitation Sciences _16.docx]

© Journal of Research in Rehabilitation Sciences . This work is licensed under CC BY-NC 4.0 (https://creativecommons.org/licenses/by-nc/4.0/).

Volume & Issue: دوره 11، شماره 1: 1394

Title: اثر یکپارچگی بینایی – شنوایی بر دقت اجرا و یادگیری یک تکلیف حرکتی

http://jrrs.mui.ac.ir/index.php/jrrs/article/view/2176

مقدمه: بینایی تنها حس فراهم‌کننده اطلاعات برای درک الگوی حرکت نیست. شنوایی کانال ادراکی مناسب دیگری برای جمع‌آوری اطلاعات درباره الگوی حرکت است.از این رو تحقیق حاضر به بررسی اثر یکپارچگی بینایی- شنوایی بر دقت اجرا و یادگیری تکلیف می‌پردازد. مواد و روش‌ها: 30 دانشجوی دوره کارشناسی دانشگاه شهید بهشتی تهران به طور در دسترس انتخاب شدند. افراد به طور تصادفی در سه گروه بینایی، بینایی-شنوایی (تک‌کانالی) و بینایی- شنوایی (دوکانالی) قرار گرفتند. افراد در گروه بینایی الگوی فرد ماهری را تماشا می‌کردند و در گروه‌های بینایی–شنوایی همزمان و علاوه بر الگوی بینایی، سرعت زاویه‌ای مفصل آرنج (گروه دوم) و سرعت زاویه‌ای مفاصل آرنج و مچ (گروه سوم) را دریافت می‌کردند. آزمودنی‌ها 4 جلسه 40 کوششی تمرین کردند و در انتهای هر جلسه، آزمون اکتساب به عمل آمد. 48 ساعت بعد، آزمون یادداری انجام گردید.برای تحلیل داده‌ها از آزمون‌های One-way ANOVA و Repeated measures ANOVA استفاده شد. تحلیل‌ها با نرم افزار SPSS و در سطح معنی داری 05/0 انجام شد. یافته‌ها: در آزمون اکتساب، گروه بینایی- شنوایی (تک‌کانالی) عملکرد بهتر معنی‌داری نسبت به گروه بینایی (001/0 P =) و بینایی- شنوایی (دوکانالی) (045/0P =) داشت. در آزمون یادداری، بین هر دو گروه بینایی- شنوایی (تک‌کانالی و دوکانالی) با گروه بینایی، تفاوت معنی‌داری به نفع گروه‌های دو حسی وجود داشت (به ترتیب، 002/0P =  و 001/0P = ). نتیجه‎گیری: نتایج این تحقیق مطابق با فرضیه تناسب حسی است و بیان می‌کند که در تکلیف شوت جفت بسکتبال به دلیل سازگاری‌های زمانی خاص، حسی که بر ادراک در زمینه تکلیف مسلط است، حس شنوایی است که اگر همراه با بینایی ارائه شود نتایج سودمندی را به دنبال دارد. به طور کلی در این تحقیق اثر مثبت یکپارچگی بینایی- شنوایی بر عملکرد و یادگیری شوت جفت بسکتبال تأیید شد.

Title: روایی و پایایی نسخه فارسی پرسشنامه ارزیابی عادات زندگی برای افراد سالمند

http://jrrs.mui.ac.ir/index.php/jrrs/article/view/2029

مقدمه: مشارکت اجتماعی ویژگی اصلی سالمندی موفق و سالم و هدف نهایی مداخلات در حوزه سلامت می باشد. این مطالعه به منظور ترجمه و تعیین پایایی و روایی پرسشنامه ارزیابی عادات زندگی (Life Habits) (فرم کوتاه)، در راستای اندازه گیری مشارکت اجتماعی سالمندان طراحی شد. مواد و روش‌ها: پس از کسب اجازه از نویسنده بر اساس روش Forward-Backward این پرسشنامه ترجمه شد و سپس روایی و پایایی آن در جمعیت 150 نفری از سالمندان شهر تهران (سن 60 سال و بالاتر)، مورد بررسی قرار گرفت. داده‌های به دست آمده از نظر روایی صوری، روایی محتوایی (روش لاوشه) و پایایی آزمون-بازآزمون (تکرار پذیری به فاصله 2 هفته) مورد بررسی قرار گرفت. یافته‌ها: روایی صوری و محتوایی [88/0 = CVI (Content validity index)] پرسشنامه براساس نظر متخصصین و سالمندان تایید شد. میزان پایایی آزمون – باز آزمون 98/0 [95/0 = ICC (Intraclass correlation coefficient)] به دست آمد. نتیجه‎گیری: مجموعه نتایج به دست آمده نشان می دهد که گونه فارسی پرسشنامه ارزیابی عادات زندگی از پایایی و روایی قابل قبولی برخوردار است. بنابراین پس از اجراي روايي سازه مي تواند به عنوان ابزار بالینی و تحقیقاتی برای بررسی مشارکت اجتماعی سالمندان بكار رود.

Title: مقايسه تأثير هشت هفته تمرينات هوازي در آب و تمرينات تعادلي بر ناتواني جسماني و سلامت رواني زنان مبتلا به مولتیپل اسکلروزیس

http://jrrs.mui.ac.ir/index.php/jrrs/article/view/2018

مقدمه: مولتیپل اسکلروزیس (Multiple sclerosis یا MS) يكي از شايع‌ترين بيماري‌هاي مزمن سيستم اعصاب مركزي است که با نشانه‌های گوناگون بالینی از جمله خستگی، ناتوانی جسمانی و ... آشکار می‌گردد. پژوهش حاضر با هدف مقايسه تأثير هشت هفته تمرينات هوازي در آب و تمرينات تعادلي بر ناتواني جسماني و سلامت رواني بيماران زن مبتلا به  MS اجرا شده است. مواد و روش‌ها: طرح تحقیقی حاضر از نوع کاربردی و روش تحقیق نیمه تجربی بوده که تعداد 45 نفر از بيماران زن مبتلا به مولتیپل اسکلروزیس عضو انجمن MS شهرستان دزفول با درجه ناتوانی جسمانی 1 تا 4، مدت بیماری 12 تا 48 ماه و دامنه سنی 20 تا 45 سال، به عنوان نمونه آماري انتخاب و به صورت تصادفي در سه گروه 15 نفری تمرینات هوازی در آب ، تمرینات تعادلی و شاهد قرار گرفتند. هر دو گروه تمرین، به مدت 8 هفته و هر هفته 3 جلسه 40 تا 50 دقیقه ای به تمرینات مربوط به خود پرداختند. آزمودنی‌ها مقياس ناتواني جسماني Kurtzke و پرسشنامه سلامت عمومي Goldberg و Hiller را  قبل و بعد از 8 هفته تكميل نمودند، داده‌ها با آزمون‌های آماری t همبسته، One-way analysis of variance (One-way ANOV) و Multivariate analysis of variance (MANOVA) و آزمون تعقیبی Tukey تجزیه و تحلیل شدند. یافته‌ها: در هر دو گروه تمرینات هوازی در آب و تمرینات تعادلی، ناتوانی جسمانی کاهش معنی دار و سلامت روانی افزایش معنی داری داشته است و تفاوت معنی داری بین دو گروه تمرینی مشاهده نشد. نتیجه‎گیری: در نهایت با توجه به یافته‌های این پژوهش می‌توان گفت، هر دو شیوه تمرینی هوازی در آب و تعادلی می‌توانند در کاهش ناتوانی جسمانی و افزایش سلامت روانی بیماران زن مبتلا به  MS تأثير  معنی داری داشته باشند.

Title: تأثير يک دوره تمرين هوازي منتخب بر شدت سردرد بيماران مبتلا به ميگرن

http://jrrs.mui.ac.ir/index.php/jrrs/article/view/2243

مقدمه: حملات ميگرن رويداد مغزي پيچيده اي است که مي‌تواند علائم عصبي و جسماني متعددي داشته باشد. اگرچه سردرد  برجسته ترین ويژگي ميگرن است، ميگرن ممکن است داراي علائم ديگري هم باشد که قبل، درحين و يا بعد از سردرد ايجاد مي‌شود. هدف اين مطالعه بررسي اثر يک دوره تمرينات هوازي منتخب روي شدت سردرد بيماران مبتلا به ميگرن بود. مواد و روش‌ها: تحقيق حاضرکاربردي و از نوع نيمه تجربي بود و نمونه‌هاي آن شامل 22 زن مبتلا به ميگرن، با ميانگين سني گروه تجربي53/6 ± 69/31 وميانگين سني گروه شاهد 30/9 ± 55/37 سال بود که به صورت در دسترس انتخاب و به شکل داوطلبانه در دو گروه تجربي(13 نفر) و شاهد(نفر9) قرارگرفتند. گروه تجربي علاوه بر درمان دارويي به مدت12 هفته، سه جلسه در هفته در تمرينات هوازي منتخب شرکت داشتند. اين تمرينات شامل دويدن روي دستگاه تردمیل، رکاب زدن روي دوچرخه ثابت به مدت 35 تا 60 دقيقه و با شدت 50 تا 75 درصد حداکثر ضربان قلب بود.گروه شاهد فقط تحت درمان دارویی بودند و هیچ برنامه ی ورزشی منظمی نداشتند. دو گروه در ابتدا و انتهای مطالعه تحت آزمون قرار گرفتند. یافته‌ها: آزمون t مستقل اختلاف بين ميانگين‌ها‌ نشان داد که 12 هفته تمرين ورزشي موجب كاهش معني داري در شدت سردرد بيماران مبتلا به ميگرن شد، و از مقدار23/34  به مقدار30/14 کاهش نشان داد. نتیجه‎گیری: يافته‌هاي تحقيق نشان مي دهد که احتمالاً مي توان از تمرينات هوازي به عنوان يک روش درماني مکمل در کنار ساير درمان‌هاي دارويي ميگرن به منظور بهبود شدت سردرد بيماران مبتلا به ميگرن استفاده کرد.

Title: اثربخشی توانبخشی شناختی بر خطاهای املا دانش آموزان با نارسا نویسی

http://jrrs.mui.ac.ir/index.php/jrrs/article/view/2118

مقدمه: ناتوانی در املا از جمله شایع ترین اختلالات یادگیری است. درمان‌های رایج برای مشکل املا کارایی چندانی ندارند. لذا در پژوهش حاضر به بررسی اثربخشی توانبخشی شناختی  بر خطاهای املا دانش‌آموزان با نارسانویسی پرداخته شد. مواد و روش‌ها: طرح مورد استفاده در این پژوهش، طرح تک آزمودنی با خط پایه چند گانه بود. نمونه پژوهشی تعداد 6 دانش‌آموز پسر مقطع دوم ابتدایی شهرستان تبریز  با هوش نرمال و دارای مشکل املا بود که مشکل بالینی در هیچ یک از خرده مقیاس‌های فهرست رفتاری کودک (Child Behavior Checklist یا CBCL) نداشتند. تعداد جلسات آموزشی 30 جلسه بود و جهت آموزش از نرم‌افزارSound Smart ، تکالیف شناختی مرکز علوم مغز و شناخت دانشگاه کمبریج و نیز تکالیف مداد- کاغذی استفاده شد. برای ارزیابی تعداد خطاهای املا از آزمون محقق ساخته املا استفاده شد. روش‌های آماری به کار رفته شامل محاسبه اندازه اثر، درصد بهبودی و تحلیل چشمی نمودارها بود. یافته‌ها: در خطاهای مربوط به مهارت دیداری، میانگین اندازه اثر برای خط پایه-درمان 6 آزمودنی برابر 03/1 (اندازه اثر متوسط) و میانگین اندازه اثر برای خط پایه- پیگیری برابر 26/2 (اندازه اثر بالا) به دست آمد. همچنین در خطاهای مربوط به مهارت شنیداری میانگین اندازه اثر برای خط پایه-درمان برای 6 آزمودنی 92/0 (اندازه اثر متوسط) و میانگین اندازه اثر برای خط پایه- پیگیری برابر 15/2 (اندازه اثر بالا) به دست آمد. درصد بهبودی خطاهای  دیداری و شنیداری املا در مرحله پیگیری در مقایسه با خط پایه  بیشتر از مرحله مداخله در مقایسه با خط پایه بود. نتیجه‎گیری: می‌توان گفت که برنامه توانبخشی شناختی می‌تواند به عنوان روشی جدید و جذاب برای کودکان، در کنار سایر روش‌های متداول برای اختلال املا با در نظر گرفتن تفاوت‌های فردی مورد استفاده قرار گیرد.

Title: تأثیر بازخورد حسی بر انتقال فاز نسبی در تکلیف هماهنگی دو دستی در سالمندان

http://jrrs.mui.ac.ir/index.php/jrrs/article/view/2146

مقدمه: هدف از اجرای این تحقیق بررسی تأثیربازخورد حسی بر سرعت انتقال فاز نسبی الگوی هماهنگی دودستی برون مرحله در میان افراد سالمند بود. مواد و روش‌ها: برای این منظور، 15 مرد سالمند سالم با دامنۀ سنی 60 تا 70 سال که همگی راست دست بودند به صورت در دسترس انتخاب شدند. شرکت‌کنندگان حرکت خود رابر روی دستگاه هماهنگی دو دستیبا فرکانس 75/0 هرتز و با الگوی هماهنگی برون مرحله شروع کرده و به تدریج هر 5 ثانیه، 25/0 هرتز به فرکانس افزوده شد تا در نهایت فرکانس 5 هرتز که انجام تکلیف خاتمه یابد. این تکلیف تحت 8 وضعیت حسی متفاوت اجرا شد. داده‌ها توسط تحلیل واریانس2×2×2 با اندازه‌گیری مکررو آزمون Paired t برای مقایسه‌های دو به دوی اثرات تعاملی با تصحیح Bonefrroni بررسی شدند. یافته‌ها: یافته‌ها نشان دادند که اثر اصلی حس عمقی (001/0 = P)، شنوایی(001/ 0=P )و اثر تعامل دوطرفۀ شنوایی-بینایی (022/0 = P) معنی‌دار بود. این یافته‌ها بر تأثیر مثبت بازخوردشنوایی و حس عمقی و همچنین تأثیر بیشتر وجود اطلاعات شنوایی در مقابل عدم وجود بینایی بر سرعت انتقال فاز نسبی دلالت کردند. نتیجه‎گیری: یافته‌ها نشان داد که اجرای تکلیف هماهنگی دو دستی حاضر تحت کنترل سیستم حلقه بسته بود. همچنین بازگوکنندۀ نقش پارامتر کنترل (فرکانس) در سرعت انتقال فاز نسبی از الگوی ناپایدار به الگوی پایدارتر با افزایش سرعت حرکت بود. از آنجایی که اجرای الگوی هماهنگی سالمندان توسطمداخلات شنوایی و حس عمقی بیشتر از مداخله بینایی تخریب شد و به دنبال آن تغییرپذیری بیشتر و در نهایت انتقال فاز سریع‌تری را نشان دادند، می‌توان نتیجه گرفت که این افراد بیشتر بر حس عمقی و شنوایی خود برای اجرای الگوی هماهنگی دو دستی متکی می‌باشند.

Title: مقایسه تاثیر سه برنامه تمرینی قدرتی، کششی و ترکیبی بر سندرم متقاطع فوقانی(UCS)

http://jrrs.mui.ac.ir/index.php/jrrs/article/view/2149

قدمه: سندرم متقاطع فوقانی (Upper cross syndrome یا UCS)نوعی از درگیری سیستم عضلانی اسکلتی می باشد که منجر به کوتاهی عضلات قدامی و ضعف عضلات خلفی می‌شود. از روش های رایج در اصلاح این ناهنجاری ها تمرینات اصلاحی می باشد. هدف از انجام این تحقیق، بررسی تاثیر سه نوع برنامه تمرینی کششی، قدرتی و ترکیبی در افراد مبتلا به سندرم متقاطع فوقانی بود. مواد و روش‌ها: ۴۰ دانشجوی دختر دارای ناهنجاری سر ، و شانه به جلو و کایفوز با میانگین سنی۷۷/۱ ± ۲۲/۲۲ سال به صورت هدفدار انتخاب شدند و به صورت تصادفی به سه گروه ۱۰ نفره تجربی و یک گروه ۱۰ نفره شاهد تقسیم شدند. گروه تجربی اول تمرینات قدرتی، گروه تجربی دوم تمرینات کششی و گروه سوم تمرینات ترکیبی را به مدت ۶ هفته انجام دادند. در این مدت گروه شاهد هیچ گونه برنامه تمرینی را دریافت نکردند. از روش عکس برداری از نمای نیمرخ برای اندازه گیری همزمان زوایای سر و شانه به جلو استفاده شد. از خط کش منعطف برای اندازه گیری میزان زاویه کایفوز آزمودنی ها استفاده شد(93/0=r). میزان تغییرات زاویه شانه، سر و کایفوز آزمودنی ها قبل و بعد از ۶ هفته تمرین اندازه گیری شد. برای تجزیه و تحلیل داده‌ها از آزمون Paired t و با استفاده از نرم‌افزار SPSS استفاده شد (05/0 ≥ P). یافته‌ها: بر اساس نتایج آزمون Paired t کاهش معنی داری در زاویه شانه به جلو (001/0 = P )، سربه جلو (005/0 = P) و کایفوز (001/0 = P) در هر سه نوع برنامه تمرینی(قدرتی، کششی، ترکیبی) بعد از ۶ هفته یافت شد. اما کاهش معنی داری در زاویه شانه به جلو، سربه جلو و کایفوز در گروه شاهد یافت نشد. تجزیه و تحلیل واریانس یکطرفه (ANOVA) نشان داد که بین تاثیر تمرینات مختلف در میزان زاویه شانه به جلو (001/0P = )، سر به جلو (001/0P = ) و کایفوز (001/0 P = ) تفاوت معنی داری وجود دارد. با توجه به نتایج آزمون تعقیبی Tukey اجرای تمرینات اصلاحی قدرتی (۰۰۱/۰P = )، کششی (۰۰۱/۰P = ) و جامع (۰۰۱/۰P = ) با گروه شاهد تفاوت معنی داری وجود دارد. درحالی که بین گروه های تمرینی قدرتی، کششی و جامع با یکدیگر در گروه شانه به جلو تفاوت معنی داری مشاهده نشده است. این در حالی است که بین تأثیر تمرینات ترکیبی در ناهنجاری سر به جلو و کایفوز در مقایسه با تمرینات دیگر تفاوت معنی داری مشاهده شد. نتیجه‎گیری: میزان کاهش زوایای سر به جلو و کایفوز در گروه ترکیبی نسبت به دو برنامه تمرینی دیگر، اثر بخشی مطلوبتر این برنامه تمرینی را نشان می دهد. با توجه به کاهش معنادار زاویه شانه و سربه جلو و کایفوز در افراد گروه تجربی، استفاده از این سه نوع برنامه تمرینی در افراد مبتلا  به عارضه UCS توصیه می‌شود.

Title: بررسی ارتباط مهار پاسخ و انعطاف پذیری شناختی با علائم رفتاری در کودکان مبتلا به اختلال اوتیسم با عملکرد بالا

http://jrrs.mui.ac.ir/index.php/jrrs/article/view/1866

مقدمه: رابطه علائم اوتیسم با توانایی‌های شناختی می‌تواند بررسی سبب شناسی و درمان این اختلال را تسهیل نماید. تخریب کارکرد اجرایی یکی از چندین فنوتیپ‌های شناختی بالقوه در اوتیسم است. لذا هدف این مطالعه بررسی رابطه مهار پاسخ و انعطاف پذیری شناختی با علائم اختلال اوتیسم با عملکرد بالا است. مواد و روش‌ها: در این پژوهش مقطعی 50 کودک مبتلا به اوتیسم با عملکرد بالا با استفاده از روش نمونه‌گیری در دسترس براساس آزمون سنجش دامنه طیف اوتیسم انتخاب شد. سپس آزمون GARS (Gilliam Autism Rating Scale) توسط درمانگران و آزمون های نوروسایکولوژیک انعطاف پذیری شناختی و مهار پاسخ توسط آزمودنی‌ها تکمیل گردید. جهت تجزیه و تحلیل داده‌ها از ضریب همبستگی Pearson و تحلیل رگرسیون چندمتغیری استفاده شد. یافته‌ها: بین مهار پاسخ با علائم ارتباطات و تعاملات اجتماعی رابطه منفی و معنی دار وجود دارد (001/0 > P ، 01/0 > P، 05/0 > P). همچنین انعطاف پذیری شناختی با علائم ارتباطات، تعاملات اجتماعی و رفتارهای کلیشه‌ای رابطه منفی و معنی دار داشت (001/0 > P، 01/0 > P، 05/0 > P). همچنین نتایج تحلیل رگرسیون نشان داد که مهار پاسخ توانایی پیش بینی علائم ارتباطات و انعطاف پذیری شناختی توانایی پیش بینی علائم تعاملات اجتماعی را دارند (05/0 > P و 01/0 > P).نتیجه‎گیری: نتایج این مطالعه حاکی از نقش مهار پاسخ و انعطاف پذیری شناختی در علائم اختلال اوتیسم با عملکرد بالا است. لذا پیشنهاد می‌شود مداخلات درمانی نوین در ترمیم مهار پاسخ و انعطاف پذیری شناختی در کودکان مبتلا به اوتیسم طراحی شود

Title: بررسی تأثیر مداخلات مختلف تمرینی بر کمر درد مزمن غیر اختصاصی: مروری بر مطالعات فارسی زبان

http://jrrs.mui.ac.ir/index.php/jrrs/article/view/1859

مقدمه: درمان محافظه کارانه بیماران کمر درد مزمن غیراختصاصی با رویکرد ورزش درمانی، جایگاه ویژه ای در توانبخشی این بیماران دارد. این مطالعه مروری با هدف بررسی تحقیقات فارسی زبان انجام گرفته در زمینه اثرات مداخلات تمرینی بر بیماران مبتلا به این عارضه انجام گرفت. مواد و روش‌ها: از موتورهای جستجوگر ISC (Islamic World Science Citation Center)، Magiran، IranMedex، SID و MedLib و کلمات کلیدی کمر درد مزمن غیراختصاصی، ورزش درمانی، حرکت درمانی، تمرین درمانی،حرکات (تمرینات) اصلاحی جهت یافتن مقالات مرتبط استفاده گردید. پژوهش‌هایی که از آزمودنی‌ها با کمر در حاد یا اختصاصی استفاده کرده بودند، از روند مطالعه کنار گذاشته شدند. یافته‌ها: از بین 35 مقاله یافت شده، تعداد 10 مقاله معیارهای ورود را کسب نمودند. این مطالعات به طور عمده به بررسی تأثیر تمرینات ثباتی مرکزی و پیلاتس بر شدت درد، میزان ناتوانی، عملکرد، تعادل، استقامت عضلات فلکسور و اکستنسور کمری، و فعالیت الکتریکی عضلات شکمی و ستون فقرات کمری در بیماران مبتلا به کمر درد مزمن غیراختصاصی پرداخته بودند. اثرات مثبت تمرینات ثباتی مرکزی و پیلاتس بر متغیرهای مذکور در این مطالعات به ثبت رسیده است. نتیجه‎گیری: ورزش درمانی؛ بویژه در قالب تمرینات ثباتی مرکزی یا پیلاتس، می تواند منجر به کاهش درد و بهبود عملکرد بیماران مبتلا به کمر درد مزمن غیراختصاصی گردد؛ اگرچه طراحی و اجرای کارآزمایی های بالینی جهت نیل به نتایج معتبرتر با توجه به کیفیت نسبتاً پایین مقالات موجود در این زمینه، ضروری به نظر می‌رسد.

Title: فاکتورهای خطر بیومکانیکی آسیب‌‌های غیرتماسی رباط صلیبی قدامی: مروری بر متون

http://jrrs.mui.ac.ir/index.php/jrrs/article/view/1892

مقدمه: آسیب رباط صلیبی قدامی زانو ACL) یا (Anterior cruciate ligament یکی از شایع‌ترین آسیب‌های ورزشی به شمار می‌آید. وضعیت مفاصل مختلف بدن، الگوی کنترل عصبی- عضلانی وعملکرد عضلات در طول فعالیت‌های ورزشی می‌تواند بر میزان نیروهای وارد بر ACLو نیز نرخ آسیب این رباط موثر باشد. هدف از این مطالعه بررسی فاکتورهای بیومکانیکی تأثیرگذار در آسیب‌های غیرتماسی ACL می‌باشد. یکی از مهمترین فاکتورهای خطر آسیب ACL، فاکتورهای بیومکانیکی هستند.وضعیت و گشتاور حول مفصل زانو و قسمت‌های دیگر بدن از جمله مفاصل هیپ و مچ پا به هنگام فعالیت‌های ورزشی می‌تواند بر میزان نیروهای وارد بر ACL موثر باشد. هم راستا با شیوع بیشتر آسیب ACL در زنان، وجود فاکتورهای خطرسازی که می تواند ACLرا در معرض آسیب بیشتری قرار دهد، در زنان نسبت به مردان بیشتر به چشم می‌خورد. بنابراین به نظر می‌رسد، استفاده از روش‌هایی همچون تمرین‌های نوروماسکولار که بتواند این فاکتورها را کنترل کند، می‌تواند به کاهش نیروهای وارد بر ACL و نرخ آسیب آن به ویژه در زنان کمک کند.
